# Good Outcome for Very High Risk Adult B-cell Acute Lymphoblastic Leukaemia Carrying Genetic Abnormalities t(4;11)(q21;q23) or t(9;22)(q34;q11), if Promptly Submitted to Allogeneic Transplantation, after Obtaining a Good Molecular Remission

**DOI:** 10.4084/MJHID.2015.041

**Published:** 2015-06-01

**Authors:** Matteo Parma, Clara Viganò, Monica Fumagalli, Federica Colnaghi, Arianna Colombo, Federica Mottadelli, Vincenzo Rossi, Elena Elli, Elisabetta Terruzzi, Angelo Belotti, Giovanni Cazzaniga, Enrico Maria Pogliani, Pietro Pioltelli

**Affiliations:** 1 Haematology Division and BMT Unit, Ospedale San Gerardo, Monza, Italy.; 2 Centro Ricerca Tettamanti, Clinica Pediatrica Università di Milano Bicocca, Ospedale San Gerardo/Fondazione MBBM, Monza, Italy.; 3 Dipartimento di Scienze della Salute, Università di Milano Bicocca.

## Abstract

**Background and Objectives:**

Acute lymphoblastic leukaemia (ALL) carrying t(9;22) or t(4;11) genetic abnormalities represents a very high risk subtype of disease (VHR-ALL). Hematopoietic stem cell transplantation (HSCT) remains the best curative option not only for t(4;11) ALL, but also for t(9;22) ALL in the tyrosin-kinase inhibitors era. In the last years, low molecular level of minimal residual disease (MRD) before HSCT was reported as one of the best favourable indexes for survival in ALL. Here we observed that even these patients can show a favourable outcome if submitted to HSCT with very low MRD.

**Methods:**

We considered 18 consecutive VHR-ALL patients eligible to HSCT. 16 of them were transplanted in first remission, as soon as possible, employing myelo-ablative conditioning regimens. Molecular MRD has been evaluated before and after HSCT.

**Results:**

Immediately before HSCT, MRD revealed: complete molecular remission (MRD^neg^) for five patients, and a level <1×10^−3^ for seven patients. 100 days after HSCT we had: MRD^neg^ for seven patients and a decrease for all the others after HSCT. After the tapering of immunosuppressive drugs, 13 patients reached the MRD^neg^ in a median time of 8 months (range 3–16). In the intention to treat analysis, 14/18 patients are alive and disease free at the date of analysis. Overall survival and event free survival is of 78% and 66% respectively, with an average follow-up of 45 months (range 6–84) since HSCT.

**Conclusion:**

Early transplantation with low MRD level seems to be correlated with a favourable outcome also in VHR-ALL.

## Introduction

Acute lymphoblastic leukaemia (ALL) carrying the t(4;11)(q21;q23) or t(9;22)(q34;q11) (Philadelphia chromosome) genetic abnormalities, associated with MLL-AF4 and the BCR-ABL fusion transcripts respectively, represents a very high risk subtype of the disease (VHR-ALL).^1^,^2^

t(4;11) ALL has a major incidence in infant and adult population.^3^ Typically it shows an early-B precursor immunophenotype (CD10 negative),^4^,^5^ and it is characterized by an extreme hyperleucocytosis upon onset. Although the rate of complete remission (CR) after the induction treatment is high (more than 90%), the occurrence of relapse and death throught the first two years is very elevated in the patients treated with the sole chemotherapy: Consequently the long term overall survival (OS) rate is low, around 20%-25%.^6^ By contrast, the efficacy of allogeneic haematopoietic stem cell transplantation (HSCT) performed in first CR is clearly superior, with a five years OS around 60%.^7^,^8^ By the way, all the authors confirm this main indication in this disease.^9^

Differently from the t(4;11) ALL, the Philadelphia chromosome positive (Ph^+^) ALL is more frequent in the adult population, with an incidence between 20–30%; whereas its incidence in the paediatric population is around 5–10%. In the tyrosin-kinase inhibitors (TKI) era, the combination of chemotherapy and TKI has drastically increased the outcome. Nowadays, in terms of complete haematologic and cytogenetic response, Ph^+^ ALL has a response rate of about 90% and OS at five years is around 40%, considering all the patients involved.^10^ Despite these improvements in non allografted patients, the OS at three years is extremely low (less than 20%) while is in patients submitted to HSCT around 50%. According to the recent analysis of UKALL/ECOG trial, the favourable prognostic impact of Imatinib is due to the generation of better conditions for HSCT; indeed patients, who did not undergo HSCT, show a 5 years OS, which is very similar to those patients treated with chemotherapy alone, in the pre-Imatinib era.^11^ Also the GETH/GITMO trial has given a favourable outcome in a cohort of 45 patients affected by Ph^+^ ALL and submitted to Umbilical cord blood transplantation, showing a 5 years OS of 44%, extended to 60% in patients in molecular remission before allograft.^12^ This outcome given, very early HSCT seems to be the primary indication in Ph^+^ ALL patients suitable for this procedure.^9^ By contrast, there are recent reports, particularly from MD Anderson Group, that underline how intensive chemotherapy associated with Imatib or Dasantinib maintenance without HSCT can lead to a good outcome and the overall survival is already similar to patients submitted to HSCT.^13^ Alternative strategies, such as monoclonal antibodies (Blinatumumab or Inotuzumab-Ozagomicin) and Chimeric Antigen Receptor Modified T-Cells (CAR T). seems to be a valid alternative in B-cells ALL, as reported in some experimental trials.^14^

Furthermore, the role of Minimal Residual Disease in ALL and its monitoring have become crucial in the last few years. MRD in ALL is highly predictive of relapse in those patients, who did not become negative after the first courses of treatment or in those patients, who became positive after having been previously negative.^15^ For this reason HSCT should be considered for all the patients who show MRD positivity (MRD+) independently from the risk assessment group at the onset. Moreover, quantitative molecular assay for MRD is very useful in predicting the post-transplant outcome of these patients: in fact patients showing MRD+ at lower levels have a better outcome compared to those with higher levels. In particular, as reported in the experience of Northern Italy Leukemia Group, 10^−3^ seems to be a real threshold in term of predicting the post-transplant evolution, since patients who underwent HSCT with MRD value of 10^−3^ or more had an extremely unfavourable outcome.^16^ All the recent reports agree with the predictive role of MRD either in patients submitted to HSCT or in patients non allografted. Also the report of MDACC confirmed that a major molecular response obtained in the first months of treatment has an excellent impact also in Ph+ALL patients not submitted to HSCT.^13^

In this report, we analyzed the outcome of patients affected by t(4;11) and Ph^+^ ALL and submitted to HSCT, by investigating the role of MRD, both before and after the allograft.

## Methods

### Patients

Hereinafter, we take into consideration a group of 18 consecutive patients affected by VHR-ALL (5 with t(4:11) ALL and 13 with Ph^+^ ALL), treated in our division between January 2010 and December 2014 and eligible for HSCT upon onset of the disease. Our intention to treat analysis excluded all the patients who, in the same period, were not eligible to HSCT for clinical or other reasons. These two categories are extremely different and not comparable, and this justify the exclusion of the last one from the analysis. Due to the fact that HSCT has been considered the gold standard for VHR-ALL and the main option proposed to these patients, we have not a control group of “not HSCT patients” with similar clinical condition at baseline. Clinical characteristics were: M/F ratio 5/13, mean age 46 years (21–65), none of them showed CNS involvement. All of them have been treated with an induction course according to an IVAP scheme: Idarubicin (12 mg/m^2^ day 1,2); Vincristine (1,4 mg/m^2^ day 1; 8; 15); Asparaginase (3000 UI/m^2^ for six administrations after day +8) and Prednisone (1 mg/Kg from day 1 to 21). Two patients died during the induction or consolidation phase; the remaining 16 continued the treatment till HSCT. After the induction, 14 patients underwent some consolidation courses while the remaining 2, affected by Ph+ ALL, continued with TKI only till HSCT. Although the consolidation programs were comprehensive of different regimens, at least one course based on high-dose Methotrexate-Cytarabine or high dose Cytarabine was given to all patients. CNS prophylaxis has been carried out with an intrathecal administration of a standard dose of Cytarabine (50 mg), Methotrexate (12,5 mg) and 6-metil-Prednisolone (40 mg) during each chemotherapy course. Imatinib has been administered in Ph^+^ patients for at least three weeks in each course regularly and has been stopped for at least one week, to prevent possible resistant mutations. 400 mg × 2/day was the target dosage of Imatinib, adjustable in those patients unable to tolerate this amount. One patient showed an early relapse during the consolidation treatment, namely a T315I mutation in the BCR-ABL gene, conferring resistance to all TKI, except for Ponatinib.^17^ Therefore, the patient has been treated with Ponatinib (45 mg once a day), obtaining a second haematological remission after two months.

After VHR-ALL was diagnosed, donor research has been immediately activated, and HSCT has been promptly performed as a donor was available. Patients, who could not find a prompt donor, continued with the intensive consolidation regimen till HSCT. 15 patients underwent HSCT upon first remission and one upon second remission. Donors were: 8 siblings related, 7 matched unrelated and one haploidentical. Six months was the average time for the transplant (range 3–12), from the onset of disease. The number of courses before HSCT goes from 1 to 7 and all the patients have been treated according to the following standard mieloablative regimens: TBI-Cyclofosfamide, Busulfan-Cyclofosfamide, Busufan-Fludarabine and Busulfan-Thiotepa-Fludarabine. Complete data about the characteristics at the onset, the treatment and the outcome are shown on Table 1.

### Laboratory Findings

MRD analysis has been performed on bone marrow samples only. The BCR-ABL fusion transcript was monitored by Real-time Quantitative PCR (RQ-PCR) as previously described, by using an ABI Prism 7900HT Fast Real-time Sequence Detection System (Life Technologies, Carlsbad, CA, USA). RQ-PCR was conducted in triplicate, with a sensibility of 10^−4^.^18^ Copies of BCR-ABL fusion transcript molecules were calculated referring to a plasmid standard curve (Ipsogen-Qiagen, Marseille, France), data were normalized using ABL as housekeeping transcript, and results were expressed as number of molecules of BCR-ABL any 10^4^ ABL molecules.

A qualitative Reverse-Transcriptase PCR (RT-PCR) analysis was employed for MLL-AF4 fusion monitoring, by a two-steps (‘nested’) PCR approach.^19^ Briefly, the nested RT-PCR consisted of a second RT-PCR round by using 1 ul of the single RT-PCR amplification. PCR products have been visualized on ethidium bromide stained agarose gels. The sensitivity of single and nested RT-PCR rounds was 10^−3^ and 10^−4^, respectively. By this semi-quantitative approach, we assumed that a patient positive after single RT-PCR had an MRD level ≥10^−3^ and a patients negative after the single RT-PCR but positive after nested RT-PCR had an MRD level lower than 10^−3^ (but not negative). Only a patient negative after nested RT-PCR has been considered as truly MRD negative.^20^

Both for BCR-ABL and for MLL-AF4, complete molecular remission is defined as an undetectable MRD level (MRD^neg^).

## Results

**Figure 1:**
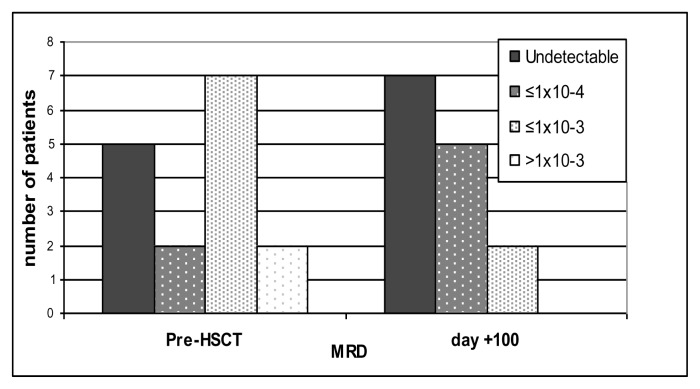
MRD levels evaluated before HSCT and 100 days after. The number of patients with MRD^neg^ or inferior to 10^−4^ (black and dark grey columns) are increasing after HSCT compared to before HSCT.

Soon before HSCT 14/16 patients showed an MRD level ≤1×10^−3^, namely: 5 patients had MRD^neg^, two patients had MRD level ≤1×10^−4^ and seven patients had an MRD level between 1×10^−4^ and 1 × 10^−3^. Only 2/16 patients showed an MRD level >1×10^−3^. 100 days after HSCT, MRD has been evaluated (MRD^+100^), in 14 patients: one patient died before because of transplant related mortality (TRM), another one has not reached 100 days yet at the time of analysis. Significantly, all 5 patients who were MRD^neg^ soon before HSCT remained MRD^neg^; as for the other ones: 2 became MRD^neg^, 5 reduced their MRD to ≤1×10^−4^ and 2 showed MRD level between 1×10^−4^ and 1×10^−3^ (Figure 1).

t(4;11) ALL patients had an extremely low MRD level or were MRD^neg^ before HSCT and all of them remained MRD^neg^ after transplantation during the entire time observation.

Ph+ ALL patients showed a different trend 100 days after HSCT: 5 patients maintained an MRD^+100^ weakly positive (≤1×10^−4^): we decided not to treat them with TKI but to try a rapid tapering of immunosuppression. The same policy has been applied to a patient with a higher MRD^+100^ (6×10^−3^) but in a reduction of 1 log compared to the MRD level before HSCT. In this way, we obtained a stable MRD^neg^ for five patients while one patient had a shorter follow-up at the time of analysis. The other patient with higher MRD^+100^ (8×10^−3^) was not treated because not immediately eligible for TKI. Unfortunately, she showed a molecular relapse after 15 months after obtaining an MRD^neg^, so she was treated therefore with Imatinib 300 mg × 2/day (maximum tolerated dosage), obtaining a second molecular remission in 3 months. The last 3 Ph+ ALL patients were MRD^neg^ at 100 days after HSCT.

**Figure 2:**
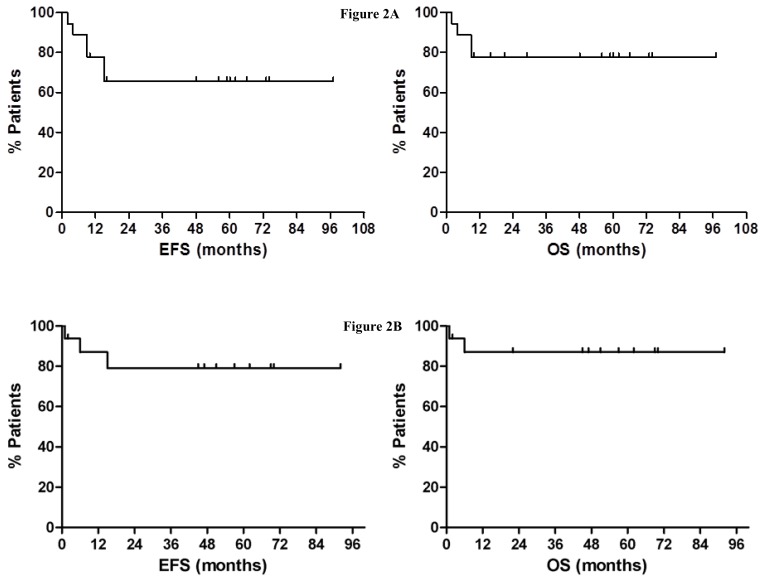
Figure 2a. EFS and OS considering all the patients. **2b.** EFS and OS considering only the patients submitted to HSCT.

Overall MRD^neg^ has been reached in 13 patients during an average time of 10 months (range 5–16); two patients had a very short follow-up at the date of analysis and 1 died. After 47 months (range 2–67) since HSCT, the outcome was as it follows. Two patients died from TRM (1 during HSCT and one for GvHD occurred six months after HSCT), a patient showed a molecular relapse 15 months after HSCT (and obtained a second molecular remission with TKI treatment), all other patients maintained the complete molecular remission. Considering that ten patients are in stable remission for more than two years, they are highly likely to be cured. Beside the two death from TRM, we also observed one patient who developed acute GvHD (grade III) followed by a JC-virus correlated encephalitis (with serious cerebral impairment): No other severe transplant-related complication has been observed during the follow-up. One bacterial pneumonia, two CMV reactivations, one haemorrhagic cystitis, one acute GvHD (grade II) and three mild chronic GvHD had a favourable outcome. After an average follow-up of 35 months (range 2–92) from HSCT, the estimated 5 years OS and event-free survival (EFS) is 78% and 66% respectively, considering all the patients (Figure 2a), but it is 86% and 79% considering the allografted patients only (Figure 2b).

## Discussion

Our experience reflects many other literature reports, which confirm that t(4;11) and Ph^+^ ALL may have a favourable outcome if patients are promptly submitted to HSCT upon first remission. In this report we took only a cohort of consecutive patients into consideration, who were suitable for HSCT, since the onset, and we intentionally excluded all the others (not eligible to HSCT), in order to evaluate the effect of a rapid transplantation upon onset of the disease. With the limitation due to the small number, we have observed that those patients, who underwent HSCT, have a high cure rate. Furthermore, we considered the role of MRD in influencing the outcome of the HSCT. Most of our patients have been allografted with a deep molecular remission, with an MRD level <1×10^−3^ and all of them, except for one, showed a favourable outcome. The only patient, who relapsed, was the only one that did not reduce again the MRD three months after HSCT. It could be hypothesized that the graft versus leukaemia effect has been less efficient in this case than in the other ones. Many reports have claimed the positive impact of TKI in Ph^+^ ALL, speculating on their role in giving better conditions for HSCT. In the TKI era, it seems therefore that Ph^+^ ALL can be allografted before, and under better state. Moreover TKI generate a deeper remission status (in terms of MRD), which is a prognostically favourable factor for the following HSCT. As previously reported in the introduction session, an MRD level <10^−3^ is a favourable prognostic index for post HSCT outcome in not VHR-ALL who fail to obtain an MRD^neg^ : we can suppose that also in Ph+ALL an MRD level below this cut-off has a favourable impact. A similar partial conclusion may be applied for t(4;11) ALL: Indeed we observed a good molecular response due to the intensive chemotherapy regimens also in this case. HSCT performed promptly and in deep molecular remission allowed a good outcome for these patients too.

In this context, a big issue is represented by all those patients who remained highly MRD+ (upper 10^−3^) independently from the risk assessment at the onset: for all of them an alternative strategy is necessary. TKI of second and third generation for Ph+ALL, nelarabine for T-ALL and monoclonal antibodies (in example Blinatumomab) for Ph negative B-ALL could be a good choice in these cases.^14^

At last, regarding Ph^+^ ALL who maintained a weakly MRD^+100^ positive (≤1×10^−4^) after HSCT, we decided not to treat them with TKI but to try a rapid tapering of immunosuppression. The goal of this strategy is to maximize the graft versus leukaemia effect avoiding other drug administration. Despite the limited positive experience here reported, in many cases the only “Graft versus Leukemia” effect seems not to be sufficient to control MRD positive in post-transplant setting. In this context, also donor lymphocytes infusion (DLI) may be considered,^21^ also if this is a debated issue, considering the immunological escape of lymphoblastic cells. Actually, in patients who remained or became MRD positive after HSCT, many alternative strategies are object of discussion and it should be taken into consideration the same options contemplated for the patients who failed to obtain a deeper molecular response before HSCT. For Ph positive ALL, the main option remains TKI, particularly of second and third generation. The limitation of TKI in this contest is the insurgence of mutations that confer resistance to the drug during the time. Ponatinib is the powerful TKI, and the only one active against T315I mutation, but also it has a limited duration in time. Monoclonal antibodies such as Blinatumumab or Inotuzumab-Ozagomicin seems to give a valid alternative particularly in Ph negative ALL. Instead CAR-T have been so far applied only in few experimental trials.

## Conclusion

This experience seems to suggest that Ph^+^ ALL and t(4;11) ALL are likely to prove a favourable outcome, if promptly submitted to HSCT, especially if the patients showing a deep remission status, in terms of molecular MRD, soon before the allograft. Moreover, a rapid tapering of immunosuppressive drugs seems to be useful in MRD^+100^ minimally positive Ph^+^ ALL patients, allowing them to avoid the TKI treatment.

Extra in-depth studies are necessary to confirm these observations.

## Figures and Tables

**Table 1:** General characteristic, treatment, MRD values and outcome of each patients included in the study.

Patients	Age/Gender Donor	Time to HSCT (months)	Disease	N° of courses Pre-HSCT	Conditioning Regimen	MRD (× 10^−4^) pre-HSCT	MRD (× 10^−4^) Day +100	Time to MRD^neg^ (months)	Complications	HSCT F-up (months)	Outcome
1	24 F MUD	8	LLA Ph+	1+ TKIs	TBI-CTX	0	0	0	-	51	Alive MRD^neg^
2	49 F Sib	3	LLA Ph+	3	Bu-Flu	0	0	0	-	45	Alive MRD^neg^
3	38 M Sib	3	LLA t(4;11)	3	TBI-CTX	0	0	0	-	57	Alive MRD^neg^
4	51 F MUD	3	LLA t(4;11)	7	Bu-Flu	0	0	0	TRM	6 †	Dead for GvHD
5	42 M MUD	5	LLA t(4;11)	4	T Bu-CY	0	0	0	Pneumonia	57	Alive MRD^neg^
6	21 F MUD	5	LLA t(4;11)	4	Bu-Flu	1	0	3	-	51	Alive MRD^neg^
7	55 F Sib	7	LLA Ph+	3	Bu-Flu	7	8	5	-	22	Alive, 2^nd^ MRD^neg^ (relapse after 15 months)
8	59 F Sib	4	LLA Ph+	1+ TKIs	BU-CY	9	0,3	5	cGvHD mild	70	Alive MRD^neg^
9	48 F Sib	4	LLA Ph+	2	TBI-CTX	55	6	8	cGvHD mild	62	Alive MRD^neg^
10	31 F MUD	5	LLA Ph+	5	TBI-CTX	1	1	16	CMV, aGvHD (III°) Encephalitis	92	Alive MRD^neg^
11	25 F MUD	12	LLA Ph+	6	Bu-Flu	2	0,4	15	CMV, Haem. Cystitis	47	Alive MRD^neg^
12	57 M Sib	4	LLA Ph+	2	TBI-CTX	7	1	12	cGvHD mild	69	Alive MRD^neg^
13	53 F Sib	9	LLA Ph+	6	BU-CY	3	0	3	aGvHD (II°)	6	Alive MRD^neg^
14	47 M Sib	8	LLA Ph+	4	TBI-CTX	9			TRM	1 †	Dead for TRM at +28
15	54 F MUD	10	LLA Ph+	6	BU-CY	9	1	nr	-	6	Alive,
16	56 F Haplo	8	LLA Ph+	4 + Ponat	Bu-Flu-TT	90	nr	nr	-	2	Alive,
17	58 M		LLA Ph+						Early death		Early Death
18	65 F		LLA t(4;11)						Early death		Early Death
